# Draft Genome Sequence of *Enterococcus canintestini* 49, a Potential Probiotic That Produces Multiple Bacteriocins

**DOI:** 10.1128/genomeA.01131-17

**Published:** 2017-10-05

**Authors:** Jeella Z. Acedo, Cherry Ibarra Romero, Sarah T. Miyata, Alysson H. Blaine, Lynn M. McMullen, John C. Vederas, Marco J. van Belkum

**Affiliations:** a Department of Chemistry, University of Alberta, Edmonton, Alberta, Canada; b CanBiocin Inc., Edmonton, Alberta, Canada; c Department of Agricultural, Food and Nutritional Science, University of Alberta, Edmonton, Alberta, Canada

## Abstract

*Enterococcus canintestini* 49, isolated from dog feces, is active against *Clostridium perfringens*, vancomycin-resistant enterococci, and *Listeria monocytogenes*. Its draft genome sequence reported herein contains a gene cluster encoding multiple bacteriocins and indicates the absence of genes for virulence factors. These characteristics signify the strain’s potential for use as a probiotic.

## GENOME ANNOUNCEMENT

Enterococci are promising sources of bacteriocins, which are ribosomally synthesized antimicrobial peptides that can be used in food biopreservation (1). In addition, some enterococci are also used as probiotics to promote human and animal health (2). However, many enterococcal strains produce virulence factors, which calls for a careful screening of strains prior to their use in biotechnological applications. *Enterococcus* sp. strain 49 was isolated from dog feces and found to inhibit the growth of *Clostridium perfringens*, vancomycin-resistant enterococci, and *Listeria monocytogenes* (data not shown). Its 16S rRNA sequence was found to be 99.9% identical to that of *Enterococcus canintestini* LMG 13590, which was also isolated from dog feces (3).

Genomic DNA of *E. canintestini* 49 was isolated using the DNeasy blood and tissue kit (Qiagen) according to the manufacturer’s instructions. The sequencing library was prepared using the Nextera XT DNA library preparation kit (Illumina), and the whole genome was sequenced using an Illumina MiSeq kit v3 (2 × 300 cycles) and MiSeq sequencing technology. The reads were assembled into contiguous sequences (contigs) using the CLC Genomics Workbench v.8 (CLC bio, Aarhus, Denmark). A sequencing coverage of 200× was attained. The assembled draft genome contains 24 contigs and 2,734,830 bases, with 36.2% GC content. The genome was annotated using the NCBI Prokaryotic Genome Automatic Annotation Pipeline (PGAAP), which predicted 2,580 genes, including 2,454 coding sequences (CDS), 7 rRNAs, and 55 tRNAs. The identity of the species was determined based on the average nucleotide identity (ANI) with previously sequenced genomes in the GenBank database as calculated by the JSpecies software (4). *E. canintestini* 49 exhibits 98.23% ANI with the *E. canintestini* DSM 21207 reference genome.

BAGEL3 (5) and antiSMASH v.3 (6) analyses of the *E. canintestini* 49 draft genome revealed the presence of a gene cluster with 96.7% nucleotide sequence identity with a bacteriocin gene cluster in *Enterococcus faecium* NKR-5-3, encoding the genes involved in the biosynthesis of enterocins NKR-5-3A, -C, -D, and -Z. Enterocins NKR-5-3A and -Z comprise a class IIb two-peptide bacteriocin, enterocin NKR-5-3C is a class IIa bacteriocin that contains a YGNGL motif, and enterocin NKR-5-3D is a bacteriocin that also acts as a bacteriocin induction peptide (7, 8). Similarly, the bacteriocin gene cluster of *E. canintestini* 49 encodes enterocins NKR-5-3A, -Z, and -D and a homolog of enterocin NKR-5-3C that differs by 1 amino acid. BAGEL3 predicted two additional putative bacteriocin genes adjacent to the enterocin NKR-5-3ACDZ homolog gene cluster in *E. canintestini* 49. Production of enterocins NKR-5-3D and -Z, and one of the additional bacteriocins predicted by BAGEL3, was detected by tandem mass spectrometry (MS/MS) sequencing of several inhibitory compounds that were isolated (data not shown). Aside from the ability of *E. canintestini* 49 to produce multiple bacteriocins, its genome sequence does not contain genes that encode virulence factors, such as surface aggregating protein, gelatinase, enterococcal surface protein, collagen-binding adhesin, and hyalurodinase. These findings highlight the potential use of *E. canintestini* 49 as a probiotic.

### Accession number(s).

This whole-genome shotgun project has been deposited in DDBJ/ENA/GenBank under the accession number LHUG00000000. The version described in this paper is version LHUG01000000.
